# Reduced occipital GABA in Parkinson disease with visual hallucinations

**DOI:** 10.1212/WNL.0000000000006007

**Published:** 2018-08-14

**Authors:** Michael J. Firbank, Jehill Parikh, Nicholas Murphy, Alison Killen, Charlotte L. Allan, Daniel Collerton, Andrew M. Blamire, John-Paul Taylor

**Affiliations:** From the Institute of Neuroscience (M.J.F., A.K., C.L.A., D.C., J.-P.T.), Newcastle University, Campus for Ageing and Vitality, Newcastle upon Tyne; and Newcastle Magnetic Resonance Centre (J.P., A.M.B.), Institute of Cellular Medicine, Newcastle University, Newcastle upon Tyne, UK; and Baylor College of Medicine (N.M.), Houston, TX.

## Abstract

**Objective:**

To investigate the relationship between visual hallucinations in Parkinson disease (PD) and levels of γ-aminobutyric acid (GABA) in the primary visual cortex.

**Methods:**

We utilized magnetic resonance spectroscopy to investigate occipital GABA levels in 36 participants with PD, 19 with and 17 without complex visual hallucinations, together with 20 healthy controls without hallucinations. In addition, we acquired T1-weighted MRI, whole-brain fMRI during a visual task, and diffusion tensor imaging.

**Results:**

We found lower GABA+/creatine in PD with visual hallucinations (0.091 ± 0.010) vs those without (0.101 ± 0.010) and controls (0.099 ± 0.010) (*F*_2,49_ = 4.5; *p* = 0.016). Reduced gray matter in the hallucinations group was also observed in the anterior temporal lobe. Although there were widespread reductions in white matter integrity in the visual hallucinations group, this was no longer significant after controlling for cognitive function.

**Conclusions:**

The data suggest that reduced levels of GABA are associated with visual hallucinations in PD and implicate changes to the ventral visual stream in the genesis of visual hallucinations. Modulation of visual cortical excitability through, for example, pharmacologic intervention, may be a promising treatment avenue to explore.

Visual hallucinations are common in Parkinson disease (PD),^1^ particularly as the disease advances, and range from relatively simple flashes of light or color to more complex hallucinations that are typically well-formed images.^2^ A number of models^3,4^ have been proposed to explain the origin of complex recurrent visual hallucinations. Unifying elements across the models include breakdown in communication between cortical regions involved in visual processing, and alteration in the weighting of internal vs external input.

A neuropathologic study of the visual system in dementia with Lewy bodies (DLB) found reduced GABAergic activity in the primary visual cortex.^5^ This may be an adaption to poor visual input or disrupted connectivity with other visual areas, with reduced GABAergic inhibition maintaining the ability to recognize objects, at a cost of seeing things that are not there.

The aims of this study were therefore to investigate γ-aminobutyric acid (GABA)+ levels using magnetic resonance spectroscopy (MRS) in the occipital lobe of patients with PD with and without complex visual hallucinations, along with similarly aged healthy participants. We also used structural, diffusion, and fMRI with a flashing checkerboard paradigm to comprehensively investigate brain changes in people with hallucinations.

We hypothesized that (1) GABA+ would be reduced in participants with visual hallucinations, and correlated with hallucination symptom severity; (2) GABA+ levels would inversely correlate with fMRI activation; and (3) in the visual system, there would be brain atrophy and disruption of white matter fibers in those with visual hallucinations, and these changes would correlate with GABA+.

## Methods

### Participants

#### Inclusion and exclusion criteria

We prospectively recruited 45 participants between 2014 and 2017 with PD aged 60 years and older, with a Mini-Mental State Examination (MMSE) score >12 from a population of local community-dwelling participants who had been referred to local neurology and old age psychiatry services. Twenty-one healthy controls were identified from spouses and friends of participants included in this and previous studies. Diagnosis of PD was made according to the UK Brain Bank criteria^6^ with any cognitive impairment either diagnosed as mild cognitive impairment according to the Movement Disorder Society level 1 criteria^7^ or dementia according to the diagnostic criteria for PD dementia.^8^ Clinical diagnoses were confirmed by an independent and experienced clinician.

Control participants in the study showed no evidence of dementia (from their history and CAMCOG [Cambridge Cognition Examination] score >80). For all participants, exclusion criteria included contraindications for MRI, history of alcohol or substance misuse, moderate to severe visual impairment, significant non-PD–related psychiatric or neurologic history, moderate to severe cerebral small vessel disease, imaging evidence of focal brain lesions, or the presence of other unstable or severe medical illness. The sample size was chosen to give 85% power to detect a 12.5% difference in GABA concentration.^9^

#### Clinical assessment

Global cognitive function was assessed using the CAMCOG and MMSE. The presence and severity of any extrapyramidal signs were graded using the motor component of the Unified Parkinson's Disease Rating Scale (UPDRS-III).

All participants had their near visual acuity measured with the Snellen chart and Landolt broken rings (test distance 40 cm) after correction of any refractive errors. Participants were excluded if they had significant visual impairment that could not be corrected. We used the best performance across all tests for each participant as a measure of acuity in group comparisons. Computerized tests of visuoperceptual function included angle and motion discrimination tasks, which have established metrics in Lewy body disease and have been reported in a number of our reports.^10,11^

As in previous work,^12^ for assessment of visual hallucinations, the hallucinations subscale of the Neuropsychiatric Inventory (NPIhall)^13^ was used with specific reference to visual hallucinations occurring in the previous month. Subsequently, we used the derived NPIhall score (frequency × severity of hallucinations) in analyses. For reliability purposes, patients and carers were independently asked about the occurrence of visual hallucinations in the month before MRI using screening questions originating from the North-East Visual Hallucinations Interview III^14^; any discrepancies in the accounts of hallucinations between patient and carer/family member were discussed with both parties and the assessor, with reformulation of NPIhall test scores (with primacy given to the opinion of the caregiver, where the patient seemed to lack insight).

We also used the noise pareidolia test^15^ as this has been shown to correlate with tendency to hallucinate in Lewy body dementia. For this test, participants were shown a series of 40 black and white images. These all contain cloud-like noise formations, and in 8 images, there is a face inserted somewhere (the location and face differ for each case). After 3 example images, participants were shown the 40 images one at a time, and asked whether they could see any faces. If so, they were asked to indicate the location of the face(s). We scored the test according to the number of illusory faces seen (i.e., faces indicated where there were not faces inserted in the image). Cognitive fluctuations were quantified with the Mayo Fluctuations Composite Score (MAYO)^16^ and the Clinician Assessment of Fluctuation scale.^17^

Participants were classed as active visual hallucinators (PD-VH) if they had complex visual hallucinations in the month preceding their interview; otherwise, they were classed as nonhallucinators (controls and PD-nonVH). Since our focus was on complex visual hallucinations, those with minor hallucinations (e.g., passage or feeling of presence) but no complex hallucinations in the last month were included in the PD-nonVH group. We made this distinction since passage and feeling of presence probably have a different etiologic basis to complex hallucinations even though minor visual hallucinations typically precede complex visual hallucinations.^18,19^

### Standard protocol approvals, registrations, and patient consents

The study was approved by the local ethics committee, and written consent was obtained from all participants (or nominated independent mental capacity advocate where participant lacked capacity).

### MRI acquisition

Participants were scanned on a 3T whole-body MR scanner (Achieva scanner; Philips Medical Systems, Best, the Netherlands) with body coil transmission and an 8-channel head coil receiver.

#### Magnetic resonance spectroscopy

**Figure 1:**
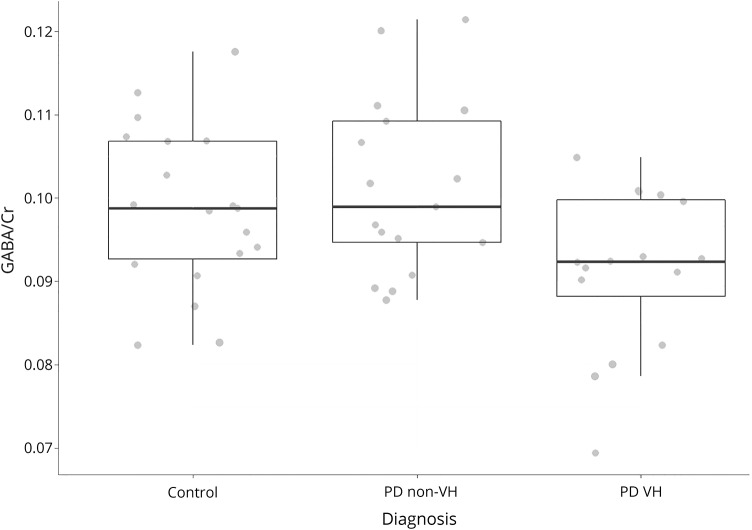
The occipital lobe GABA+/Cr ratio in the 3 groups Cr = creatine; GABA = γ-aminobutyric acid; PD = Parkinson disease; VH = visual hallucinator.

We used the MEGA-PRESS technique,^20^ as previously reported^21^ with a sinc gaussian editing pulse applied alternately at 1.9 ppm (EDIT-ON) and 7.5 ppm (EDIT-OFF). Subtraction of the EDIT-OFF from EDIT-ON spectra allows the 3-ppm GABA+ signal to be separated from the overlying creatine peak. MEGA-PRESS spectra were acquired from a voxel sized 45 × 32 × 20 mm centered on the midline of the occipital lobe (data available from Newcastle University e-prints [figure 1]: eprint.ncl.ac.uk/247552). Sequence parameters were as follows: repetition time (TR) = 2,000 milliseconds (ms); echo time (TE) = 68 ms; 320 averages; acquisition bandwidth = 1,000 Hz; VAPOR (variable power radiofrequency pulses with optimized relaxation delays) water suppression.^22^ Macromolecular suppression editing^23^ was not performed, and thus our results are of GABA+ (i.e., GABA plus macromolecules). The magnetic resonance GABA signal is thought to reflect concentrations of metabolic GABA and levels of ambient extracellular GABA that contribute to tonic GABAergic activity.^24^

#### Structural and functional magnetic resonance

We acquired images including a whole-brain structural 3-dimensional MPRAGE (magnetization-prepared rapid-acquisition gradient echo) scan with sagittal acquisition, slice thickness 1.0 mm; in-plane resolution 1.0 × 1.0 mm; TR = 8.3 ms; TE = 4.6 ms; flip angle = 8°; and SENSE factor = 2. fMRI data were collected with a gradient-echo echo planar imaging sequence (TR = 1.92 seconds; TE = 40 ms; field of view 192 × 192 mm^2^; 64 × 64 matrix size; flip angle 90°; 27 slices; slice thickness 3 mm; slice gap 1 mm) with 100 volumes (192 seconds) as participants looked at the checkerboard stimulus.

Diffusion tensor imaging (DTI) acquisitions utilized a 2-dimensional spin-echo, echo planar imaging diffusion-weighted sequence with 59 slices: TR = 6,100 ms; TE = 70 ms; flip angle = 90°; field of view = 270 × 270 mm; pixel size = 2.1 × 2.1 mm; slice thickness = 2.1 mm. Diffusion weighting was applied in 64 uniformly distributed directions (diffusion b = 1,000 s·mm^−2^) and there were 6 acquisitions with no diffusion weighting (b = 0 s·mm^−2^). We also collected an identical image with b = 0 s·mm^−2^ but with the phase encoding direction reversed for distortion correction purposes.

#### fMRI stimulus presentation

fMRI was performed with the same checkerboard paradigm as we have previously utilized.^25^ fMRI-compatible goggles with lenses that ranged from −4.0 to 4.0 diopters (0.5 increment) were used to correct any refractive errors that participants had.

The stimulus presentation was controlled by the psychophysics toolbox^26^ (psychtoolbox.org/) extension for MATLAB (MathWorks, Natick, MA). A block design was used with a full field circular checkerboard stimulus consisting of five 19.2-second blocks of a black-and-white checkerboard (inverting at 7.5 Hz) alternating with five 19.2-second baseline blocks of a gray screen. Participants were asked to focus on a central cross-hair.

### Magnetic resonance analysis

#### Spectroscopy data analysis

GABA+ quantification was performed using the Gannet toolbox for MATLAB^27^ and consisted of the following steps: (1) alignment of each pair (EDIT-ON and EDIT-OFF) of spectra^28^; (2) subtraction of aligned spectra to produce GABA+ spectra, followed by averaging across acquisitions; (3) fitting a gaussian to the 3-ppm GABA+ peak to quantify GABA+ based on the area under the curve. For a typical edited spectrum and Gaussian fit, see data available from Newcastle University e-prints (figure 1): eprint.ncl.ac.uk/247552.

Choline, creatine, and NAA (*N*-acetylaspartate) amplitudes were quantified from nonedited spectra only using the AMARES (Advanced Method for Accurate, Robust, and Efficient Spectral fitting of MRS data with use of prior knowledge) algorithm from jMRUI (java-based magnetic resonance user interface).^29^ GABA+ and NAA were expressed as ratios and normalized to creatine. MRS fit quality was assessed by an experienced physicist as described previously.^21^

#### fMRI analysis

Imaging data were processed with Statistical Parametric Mapping (SPM)12 (fil.ion.ucl.ac.uk/spm/) similar to our previous work.^25^ For each participant, the T1 anatomical image was segmented and spatially normalized in SPM using the default parameters. The fMRI data for each stimulus condition were slice timing corrected, motion corrected by aligning all functional images together, and then coregistered with the T1 anatomical image. The spatial normalization parameters from the T1 segmentation were used to transform the fMRI data to standard space with a voxel size of 3 × 3 × 3 mm. The normalized images were then smoothed with a 6 × 6 × 6 mm full width at half maximum gaussian kernel. A high-pass filter of 128 seconds was used, and serial correlations were removed with SPM's AR(1) model.

The general linear model in SPM was used to conduct a whole-brain analysis of the fMRI data. We created a design matrix by convolving the time course of the checkerboard block with the canonical hemodynamic response function and its first derivative. The 6 parameters from the motion correction were included in the design matrix as covariates of no interest. Individual participant and second-level (random-effects) group analyses were conducted. Contrast images were generated from β estimates for the comparison of checkerboard vs baseline. Results are shown with a voxelwise threshold of *p* < 0.001 (uncorrected) followed by clusterwise threshold of *p* < 0.05 family-wise error (FWE)-corrected for multiple comparisons.

We also used a region of interest (ROI) analysis focusing on the visual areas. Five ROIs in MNI (Montreal Neurological Institute) space were defined averaging across left and right hemispheres: V1, V2, V3, V4, and V5 were taken from the SPM Anatomy toolbox (fz-juelich.de/inm/inm-1/DE/Forschung/_docs/SPMAnatomyToolbox/SPMAnatomyToolbox_node.html). We also defined 3 ROIs from the overall activation across all participants—all voxels with activation in the occipital lobe, all voxels with associated deactivation (both with voxelwise threshold at *p* < 0.05 FWE-corrected), and a bilateral LGN (lateral geniculate nucleus) region (voxelwise *p* < 0.001 uncorrected).

### Structural MRI analysis

#### Gray matter

For analysis of gray matter atrophy, the T1-weighted structural images were segmented with the SPM12 segment tool, and then processed using the DARTEL (Diffeomorphic Anatomical Registration Through Exponentiated Lie Algebra) Toolbox to create a group-specific template, to which the individual images were spatially normalized. Images were modulated to preserve the total tissue amount during normalization and smoothed with an 8-mm gaussian filter. We used the SPM Anatomy toolbox^30^ to identify location of significant clusters. For each participant, we also extracted the fraction of gray matter, white matter, and CSF within the individual spectroscopy voxel location.

#### Diffusion white matter

DTI data were processed using FSL (fsl.fmrib.ox.ac.uk/fsl/fslwiki) using the topup program to correct susceptibility-induced distortions using the 2 b = 0 s·mm^−2^ images with opposite phase encoding. The eddy package was then used to correct images for eddy current distortion, movement, and motion-induced signal dropout. Fractional anisotropy (FA) and mean diffusivity (MD) were then calculated with the dtifit software, and the TBSS (tract-based spatial statistics) package^31^ used to align the FA images all together, create a white matter skeleton of major tracts, and extract FA and MD values for each participant on the white matter skeleton. The images were visually inspected at each stage.

### Statistical analysis

ROI data and clinical variables were analyzed with the Statistical Package for Social Sciences (SPSS version 19; IBM Corp., Armonk, NY). Independent *t* tests or analysis of variance was used to compare groups for continuous variables. Spearman rank correlation coefficient was used to compare continuous variables.

For the fMRI voxelwise data, a 3-group analysis of variance was performed using SPM to determine overall activation patterns and investigate group differences. Voxel-based morphometry (VBM) was done using SPM on the smoothed modulated gray matter images using a 3-group analysis of covariance, with age and intracranial volume (sum of CSF, gray matter, and white matter) as covariates, and also with the addition of CAMCOG as a measure of cognitive function. We investigated, with SPM, the relationship of GABA+/creatine (Cr) with voxelwise gray matter volume, with covariates of age, intracranial volume, and group (PD-nonVH, PD-VH, and controls).

For the diffusion analysis, voxelwise differences in MD and FA between groups (with age as a covariate) were estimated using the FSL randomise package. We also used this to look at voxelwise correlations with occipital GABA+/Cr, controlling for age and group (PD-nonVH, PD-VH, and control). This was repeated with the addition of CAMCOG as a measure of cognitive function.

### Data availability

Anonymized data on which this article is based will be shared on request with any appropriately qualified investigator.

## Results

MRI scans were obtained on 20 of the controls and 36 participants with PD, of whom 15 had mild cognitive impairment and 21 had PD dementia. Table 1 shows the demographics for these participants. There were no significant differences in age or sex between hallucination groups, and there were no differences in duration of PD or levodopa dose between the PD-VH and PD-nonVH groups. However, the PD-VH group had worse motor function according to the UPDRS-III score (*p* < 0.001), worse cognition on the CAMCOG scale (*p* < 0.001), and were more likely to be taking cholinesterase inhibitors (*p* = 0.025). As expected, the PD-VH group had significantly higher hallucination scores and were more likely to have misperceptions on the pareidolia test compared to the PD-nonVH group. Data available from Newcastle University e-prints (table 1: eprint.ncl.ac.uk/247552) compare the patients with PD with vs without dementia. There were no significant differences in age, sex, years of education, duration of PD, or levodopa dose. The participants with dementia had a higher UPDRS-III score, poorer vision, and a greater tendency to hallucinate as indicated by the Neuropsychiatric Inventory (NPI) and pareidolia test.

**Table 1:**
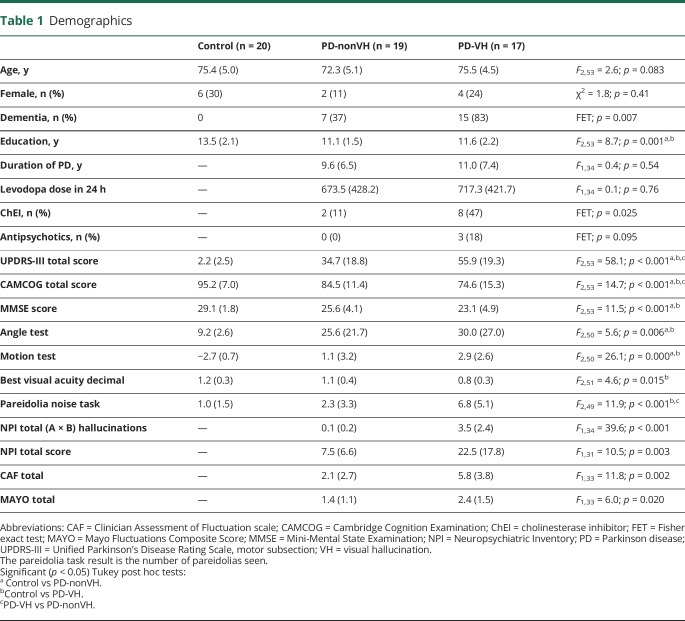
Demographics

We excluded 4 participants (1 PD-VH, 2 PD-nonVH, 1 control) from the spectroscopy analysis because they did not meet MRS quality-assurance criteria.^21^ There were no significant differences in age, sex, or diagnosis of dementia between the excluded and nonexcluded participants. Table 2 shows the ratio of GABA+ and NAA to Cr for the groups. There was a significant group difference in the GABA+/Cr ratio (figure 1), with post hoc Tukey test finding GABA+/Cr reduced in PD-VH relative to PD-nonVH. The group difference remained significant after including CAMCOG as a measure of cognitive ability in the linear model (*F*_2,48_ = 3.27, *p* = 0.047). There were no significant differences in gray or white matter proportion within the voxel between groups (table 2). Within the PD group, GABA+/Cr correlated with visual acuity (Spearman ρ = 0.4, *p* = 0.025), MMSE (ρ = 0.35, *p* = 0.047), UPDRS-III (ρ = −0.345, *p* = 0.049), and MAYO total score (ρ = −0.627, *p* < 0.001). There were no significant correlations (*p* > 0.1) with disease duration, CAMCOG, angle or motion test, or the pareidolia test. There were no significant correlations between GABA+/Cr and NPI hallucination score after controlling for VH group.

**Table 2:**
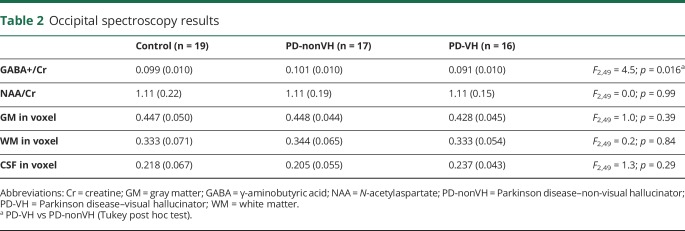
Occipital spectroscopy results

There was no significant difference in GABA+/Cr levels between participants with PD taking cholinesterase inhibitors vs those not taking them (0.098 SD 0.012 vs 0.093 SD 0.008; *t*_31_ = 1.16, *p* = 0.26), between those taking antipsychotic agents (quetiapine) vs those not (0.088 SD 0.007 vs 0.097 SD 0.011; *t*_31_ = 1.42, *p* = 0.17), and there was no significant correlation between levodopa dose and GABA+/Cr (ρ = 0.018, *p* = 0.9).

**Figure 2:**
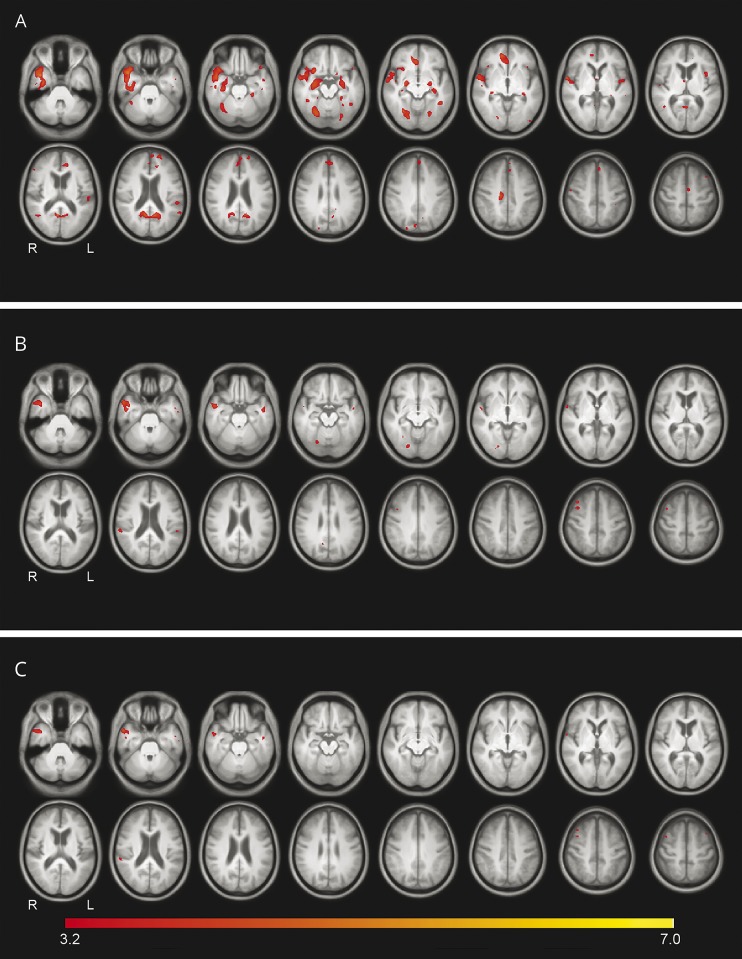
Voxelwise morphometry results, showing regions of altered gray matter Decreased gray matter for (A) PD-VH < control, (B) PD-VH < PD-nonVH, (C) PD-VH < PD-nonVH controlling for CAMCOG score. Voxelwise threshold = *p* < 0.001, uncorrected for multiple comparisons (radiologic convention: L = R). CAMCOG = Cambridge Cognition Examination; PD-nonVH = Parkinson disease–non-visual hallucinator; PD-VH = Parkinson disease–visual hallucinator.

The fMRI scans were not acquired on one PD participant, who did not tolerate the full scanning session, and 4 PD participants' scans were excluded because of excessive motion, leaving 17 PD-VH, 14 PD-nonVH, and 20 controls with usable fMRI data. All groups showed a typical activation pattern to the checkerboard (data available from Newcastle University e-prints [figure 2]: eprint.ncl.ac.uk/247552), but there were no significant differences in activation between any groups. In the ROI analysis (table 3), there were significant within-group activations in all regions apart from the V5 in the PD-VH group (1-sample test, *T*_16_ = 0.64; *p* = 0.53). However, there were no significant differences in activation between groups for any region. In the PD group, there was a significant positive correlation between the GABA+/Cr ratio and activation in the V5 ROI (Pearson degrees of freedom = 29, *r* = 0.373, *p* = 0.046) but not with the V1–V4 ROIs (Pearson *r* < 0.33, *p* > 0.08).

**Table 3:**
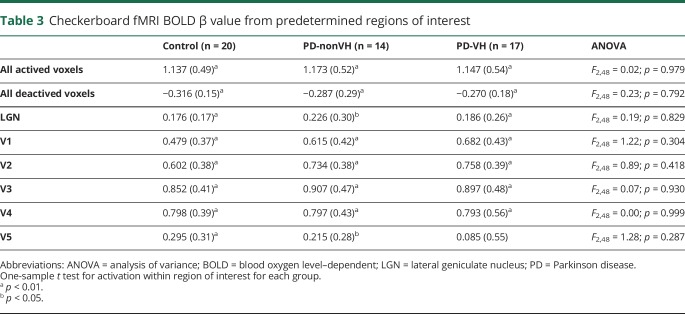
Checkerboard fMRI BOLD β value from predetermined regions of interest

MRI diffusion data were obtained on 17 PD-VH, 18 PD-nonVH, and 20 controls. The TBSS analysis found widespread differences between controls and PD-VH in both FA and MD (data available from Newcastle University e-prints [figure 3]: eprint.ncl.ac.uk/247552). However, after including CAMCOG as a covariate in the analysis, this obviated significant group differences. For the voxelwise correlations between GABA+/Cr and both MD and FA controlling for age and group, there was only a very small cluster (24 voxels) in the posterior corpus callosum. This was still significant after inclusion of CAMCOG in the model.

The VBM analysis on the 17 PD-VH, 19 PD-nonVH, and 20 controls found a significant cluster of reduced gray matter in the right anterior temporal lobe of the PD-VH group compared to both the PD-nonVH and the control group (figure 2; data available from Newcastle University e-prints [table 2]: eprint.ncl.ac.uk/247552). This cluster extended to the hippocampus and amygdala in the control vs PD-VH comparison, and there was a nonsignificant cluster in the right hippocampus and amygdala. There was also a cluster of reduced gray matter in the PD-VH compared to the control group in the V4 region (27% V4, 26% fusiform gyrus FG1, 16% V3v). With the addition of CAMCOG as a covariate to the model, there were still significant differences in the anterior temporal lobe for the PD-nonVH vs PD-VH comparison (figure 2; data available from Newcastle University e-prints [table 2]: eprint.ncl.ac.uk/247552).

To investigate associations between GABA+ and atrophy, we performed a VBM analysis of gray matter against GABA+/Cr controlling for age and group. There was an occipital cluster (66% in V1 and 22% in V2) where GABA+/Cr positively correlated with gray matter (data available from Newcastle University e-prints [table 2, figure 4]: eprint.ncl.ac.uk/247552), but this was not significant after correcting for multiple comparisons (cluster *p* = 0.08, FWE-corrected).

## Discussion

We found reduced levels of GABA+ in the PD-VH group, and there was evidence of gray matter loss in the anterior temporal lobe as well as region V4 of the visual cortex. There were, however, no alterations in functional activity in response to visual excitation by the checkerboard stimulus or in white matter diffusion parameters, once covariates were accounted for.

As hypothesized, GABA+ concentration was reduced in PD-VH compared to PD-nonVH. This agrees with the neuropathologic study finding reduced GABAergic markers in DLB.^5^ We found that the participants with hallucinations had worse acuity, which correlated with GABA+ levels in the PD group. Combined with previous research that found that occipital GABA levels decrease after eye occlusion,^32^ our findings support the hypothesis that poor input to the visual cortex leads to levels of inhibitory GABA being reduced to optimize visual processing, at the price of increased misclassifications of ambiguous stimuli.^33^ The absence of associations between GABA+ and severity of visual hallucinations suggests that low GABA levels may predispose people to hallucinate, but the occurrence of visual hallucinations is controlled by other factors, including attention and the visual environment. If visual hallucinations are partly facilitated by decreased levels of GABA in the occipital cortex causing hyperexcitability, one therapeutic strategy might be to utilize antiepileptic drugs. In PD, the 5-hydroxytryptamine type 3 (5-HT_3_) antagonist ondansetron and the 5-HT_2_ reverse agonist pimavanserin have been used to treat visual hallucinations.^34^ Since 5-HT receptors can modulate release of GABA,^35^ it may be that the mode of action of these drugs in treating hallucinations is partly through their effect on GABA.

There was no difference in functional activation between the PD-VH group and any other group. This relative lack of difference in functional activity fits with the suggestion that visual hallucinations are a side effect of neural changes aimed at preserving visual function in the face of worsening visual input or connectivity.^36^ It is also in agreement with the postmortem observations^5^ of little Lewy body disease pathology in the primary visual cortex but alterations of neurone function in the fusiform gyrus.^37^ The PD-VH group, unlike the PD-nonVH group, did not show significant activation in the V5 region. Duann et al.^38^ reported that activation in the V5 region to a flickering checkerboard was more variable within-subject compared to primary visual cortex and speculated that this might be due to differing levels of top-down influence such as paying attention to the motion aspect of the stimulus. We previously reported^25^ reduced activation in V5 in DLB to a motion stimulus, and it could be that dysfunction of this region contributes to visual hallucinations as object motion is improperly tracked, leading to discrepancies between the internal model of the world and reality.

We found only a weak association in the PD group between GABA+ and blood oxygen level–dependent (BOLD) activation in the V5 ROI. This goes against our hypothesized negative relationship between GABA and occipital BOLD activations, which was based on previous observations that these factors are related.^39^ However, some recent studies in normal participants have also failed to demonstrate a significant association between occipital GABA and BOLD.^40^ Possible explanations for the lack of an association include the fact that the BOLD signal is an indirect measure of neuronal activity and is dependent on blood flow and vascular reactivity, which could be altered in our participants.

We found widespread alterations in MD and FA in the PD-VH group in comparison to controls controlling for age. However, after including CAMCOG score in the model, there were no significant group differences, and there was only a very small region where GABA+ correlated with FA, and none with MD. Previous reports have found widespread reductions in FA and increases in MD in PD dementia,^41^ suggesting that the DTI group differences were driven by overall disease severity, rather than being specifically related to the presence of visual hallucinations. Few studies have investigated the relationship between DTI measures and visual hallucinations in PD; Lee et al.^42^ found increased MD in the parietotemporal region of PD-VH, with more widespread changes in those with dementia. Although we found that differences in MD and FA were not specifically related to visual hallucinations, nevertheless, it is possible that the presence of the white matter alterations may have contributed to the formation of hallucinations in the group, as suggested by the disconnection models of visual hallucinations.^3,4^

The ventral visual stream is likely to be involved in visual hallucination genesis, since it is chiefly responsible for object representation and recognition.^43^ The ventral stream includes projections from the primary visual cortex to the temporal lobe. Previous MRI studies of gray matter atrophy in PD-VH have found a number of regions involved, including the temporal lobe and lateral occipital lobe.^44,45^ Ventral stream temporal areas contain relatively high numbers of Lewy bodies,^46,47^ with a gradient of increasing density toward the anterior temporal lobe,^37^ and it has been speculated that these pathologic changes may contribute to visual hallucinations in DLB. The midline occipital lobe is relatively spared in DLB,^46,48^ and as shown by our fMRI data, is functionally intact, suggesting that the observed GABA reduction may be driven by ventral stream pathology leading to altered connectivity between the primary visual cortex and higher visual areas.

Our most significant structural finding was gray matter loss in the temporal pole and amygdala, along with reductions in PD-VH relative to controls in area V4 of the occipital lobe. The V4 area projects to the parahippocampal gyrus^43^ and is involved in object recognition and coordinating signals between the early and higher visual areas. The combination of atrophy in ventral stream structures, and white matter changes including to the temporal and frontal lobes, is consistent with the hypothesis^49^ of disrupted communication between the ventral visual stream and lateral frontal cortex as being mechanistically involved in the generation of visual hallucinations.

Although we used a well-established MRS technique for investigating GABA, there are some limitations to the study. The magnetic resonance spectrum of GABA is complex and coincides with that of other molecules. To maximize the signal-to-noise ratio of the MRS, we did not use macromolecule suppression techniques,^23^ and our measured signal thus represents a combination of GABA and macromolecules. Other limitations of the study include that, because of time constraints, we acquired a spectrum from only one location, and thus we are not able to say whether the GABA+ changes in PD-VH are specific to the occipital lobe. Since visual hallucinations are more common in more severe disease and in those with cognitive impairment, it may be that GABA+ levels related to disease severity, rather than specifically hallucinations. However, our finding of increased GABA+ remained significant after including a measure of global cognition in the model, suggesting that the changes were not purely driven by disease stage.

Finally, an inherent difficulty in investigating visual hallucinations is that the investigator must rely on subjective reports from the participant, thus risking misclassification, particularly in individuals with cognitive impairment, and making it more challenging to find correlates of hallucination severity. We cross-checked hallucination reports between participants and their informants to increase reliability, and used the previously validated pareidolia test,^15^ finding significantly increased rates of visual misperception in the PD-VH group, providing confidence in our visual hallucinations group classification.

We found alterations to GABA+ in the occipital cortex, together with structural changes in the ventral stream of patients with PD who had visual hallucinations. Further longitudinal studies are required to elucidate the connection between these changes and how they influence the development of visual hallucinations. This may have important translational implications, as remediation of GABAergic function or reduction in visual cortical hyperexcitability may represent a novel treatment approach for visual hallucinations in PD.

## Glossary

BOLD: blood oxygen level–dependent
CAMCOG: Cambridge Cognition Examination
Cr: creatine
DLB: dementia with Lewy bodies
DTI: diffusion tensor imaging
FA: fractional anisotropy
FWE: family-wise error
GABA: γ-aminobutyric acid
5-HT: 5-hydroxytryptamine
MAYO: Mayo Fluctuations Composite Score
MD: mean diffusivity
MMSE: Mini-Mental State Examination
MRS: magnetic resonance spectroscopy
NAA: *N*-acetylaspartate
NPI: Neuropsychiatric Inventory
NPIhall: Neuropsychiatric Inventory, hallucinations subscale
PD: Parkinson disease
PD-nonVH: Parkinson disease–non-visual hallucinator
PD-VH: Parkinson disease–visual hallucinator
ROI: region of interest
SPM: Statistical Parametric Mapping
TBSS: tract-based spatial statistics
TE: echo time
TR: repetition time
UPDRS-III: Unified Parkinson's Disease Rating Scale, motor subsection
VBM: voxel-based morphometry

## References

[1] Hely MA, Reid WG, Adena MA, Halliday GM, Morris JG. The Sydney multicenter study of Parkinson's disease: the inevitability of dementia at 20 years. Mov Disord 2008;23:837–844. 18307261 10.1002/mds.21956

[2] Urwyler P, Nef T, Muri R, et al. Visual hallucinations in eye disease and Lewy body disease. Am J Geriatr Psychiatry 2016;24:350–358. 26796926 10.1016/j.jagp.2015.10.007

[3] Muller AJ, Shine JM, Halliday GM, Lewis SJ. Visual hallucinations in Parkinson's disease: theoretical models. Mov Disord 2014;29:1591–1598. 25154807 10.1002/mds.26004

[4] Tsukada H, Fujii H, Aihara K, Tsuda I. Computational model of visual hallucination in dementia with Lewy bodies. Neural Netw 2015;62:73–82. 25282547 10.1016/j.neunet.2014.09.001

[5] Khundakar AA, Hanson PS, Erskine D, et al. Analysis of primary visual cortex in dementia with Lewy bodies indicates GABAergic involvement associated with recurrent complex visual hallucinations. Acta Neuropathol Commun 2016;4:66. 27357212 10.1186/s40478-016-0334-3 PMC4928325

[6] Hughes AJ, Daniel SE, Kilford L, Lees AJ. Accuracy of clinical diagnosis of idiopathic Parkinson's disease: a clinico-pathological study of 100 cases. J Neurol Neurosurg Psychiatry 1992;55:181–184. 1564476 10.1136/jnnp.55.3.181 PMC1014720

[7] Litvan I, Goldman JG, Tröster AI, et al. Diagnostic criteria for mild cognitive impairment in Parkinson's disease: Movement Disorder Society Task Force guidelines. Mov Disord 2012;27:349–356. 22275317 10.1002/mds.24893 PMC3641655

[8] Emre M, Aarsland D, Brown R, et al. Clinical diagnostic criteria for dementia associated with Parkinson's disease. Mov Disord 2007;22:1689–1707. 17542011 10.1002/mds.21507

[9] O'Gorman RL, Michels L, Edden RA, Murdoch JB, Martin E. In vivo detection of GABA and glutamate with MEGA-PRESS: reproducibility and gender effects. J Magn Reson Imaging 2011;33:1262–1267. 21509888 10.1002/jmri.22520 PMC3154619

[10] Wood JS, Firbank MJ, Mosimann UP, et al. Testing visual perception in dementia with Lewy bodies and Alzheimer disease. Am J Geriatr Psychiatry 2013;21:501–508. 23567415 10.1016/j.jagp.2012.11.015

[11] Firbank M, Kobeleva X, Cherry G, et al. Neural correlates of attention-executive dysfunction in Lewy body dementia and Alzheimer's disease. Hum Brain Mapp 2016;37:1254–1270. 26705763 10.1002/hbm.23100 PMC4784171

[12] Taylor JP, Firbank M, Barnett N, et al. Visual hallucinations in dementia with Lewy bodies transcranial magnetic stimulation study. Br J Psychiatry 2011;199:492–500. 22016436 10.1192/bjp.bp.110.090373 PMC3227808

[13] Cummings JL. The Neuropsychiatric Inventory: assessing psychopathology in dementia patients. Neurology 1997;48:S10–S16. 10.1212/wnl.48.5_suppl_6.10s 9153155

[14] Mosimann UP, Collerton D, Dudley R, et al. A semi-structured interview to assess visual hallucinations in older people. Int J Geriatr Psychiatry 2008;23:712–718. 18181237 10.1002/gps.1965

[15] Yokoi K, Nishio Y, Uchiyama M, Shimomura T, Iizuka O, Mori E. Hallucinators find meaning in noises: pareidolic illusions in dementia with Lewy bodies. Neuropsychologia 2014;56:245–254. 24491313 10.1016/j.neuropsychologia.2014.01.017

[16] Ferman TJ, Smith GE, Boeve BF, et al. DLB fluctuations: specific features that reliably differentiate DLB from AD and normal aging. Neurology 2004;62:181–187. 14745051 10.1212/wnl.62.2.181

[17] Walker MP, Ayre GA, Cummings JL, et al. The Clinician Assessment of Fluctuation and the One Day Fluctuation Assessment Scale:two methods to assess fluctuating confusion in dementia. Br J Psychiatry 2000;177:252–256. 11040887 10.1192/bjp.177.3.252

[18] Archibald NK, Clarke MP, Mosimann UP, Burn DJ. Visual symptoms in Parkinson's disease and Parkinson's disease dementia. Mov Disord 2011;26:2387–2395. 21953737 10.1002/mds.23891

[19] Ffytche DH, Creese B, Politis M, et al. The psychosis spectrum in Parkinson disease. Nat Rev Neurol 2017;13:81–95. 28106066 10.1038/nrneurol.2016.200 PMC5656278

[20] Mescher M, Merkle H, Kirsch J, Garwood M, Gruetter R. Simultaneous in vivo spectral editing and water suppression. NMR Biomed 1998;11:266–272. 9802468 10.1002/(sici)1099-1492(199810)11:6<266::aid-nbm530>3.0.co;2-j

[21] Sedley W, Parikh J, Edden RA, Tait V, Blamire A, Griffiths TD. Human auditory cortex neurochemistry reflects the presence and severity of tinnitus. J Neurosci 2015;35:14822–14828. 26538652 10.1523/JNEUROSCI.2695-15.2015 PMC4635131

[22] Tkáč I, Starčuk Z, Choi IY, Gruetter R. In vivo 1H NMR spectroscopy of rat brain at 1ms echo time. Magn Reson Med 1999;41:649–656. 10332839 10.1002/(sici)1522-2594(199904)41:4<649::aid-mrm2>3.0.co;2-g

[23] Edden RA, Puts NA, Barker PB. Macromolecule-suppressed GABA-edited magnetic resonance spectroscopy at 3T. Magn Reson Med 2012;68:657–661. 22777748 10.1002/mrm.24391 PMC3459680

[24] Dyke K, Pépés SE, Chen C, et al. Comparing GABA-dependent physiological measures of inhibition with proton magnetic resonance spectroscopy measurement of GABA using ultra-high-field MRI. Neuroimage 2017;152:360–370. 28284797 10.1016/j.neuroimage.2017.03.011 PMC5440178

[25] Taylor JP, Firbank MJ, He J, et al. Visual cortex in dementia with Lewy bodies: magnetic resonance imaging study. Br J Psychiatry 2012;200:491–498. 22500014 10.1192/bjp.bp.111.099432 PMC3365275

[26] Brainard DH. The Psychophysics Toolbox. Spat Vis 1997;10:433–436. 9176952

[27] Edden RA, Puts NA, Harris AD, Barker PB, Evans CJ. Gannet: a batch-processing tool for the quantitative analysis of gamma-aminobutyric acid-edited MR spectroscopy spectra. J Magn Reson Imaging 2014;40:1445–1452. 25548816 10.1002/jmri.24478 PMC4280680

[28] Near J, Edden R, Evans CJ, Paquin R, Harris A, Jezzard P. Frequency and phase drift correction of magnetic resonance spectroscopy data by spectral registration in the time domain. Magn Reson Med 2014;73:44–50. 24436292 10.1002/mrm.25094 PMC5851009

[29] Naressi A, Couturier C, Castang I, de Beer R, Graveron-Demilly D. Java based graphical user interface for MRUI, a software package for quantitation of in vivo/medical magnetic resonance spectroscopy signals. Comput Biol Med 2001;31:269–286. 11334636 10.1016/s0010-4825(01)00006-3

[30] Eickhoff SB, Stephan KE, Mohlberg H, et al. A new SPM toolbox for combining probabilistic cytoarchitectonic maps and functional imaging data. Neuroimage 2005;25:1325–1335. 15850749 10.1016/j.neuroimage.2004.12.034

[31] Smith SM, Jenkinson M, Johansen-Berg H, et al. Tract-based spatial statistics: voxelwise analysis of multi-subject diffusion data. Neuroimage 2006;31:1487–1505. 16624579 10.1016/j.neuroimage.2006.02.024

[32] Lunghi C, Emir UE, Morrone MC, Bridge H. Short-term monocular deprivation alters GABA in the adult human visual cortex. Curr Biol 2015;25:1496–1501. 26004760 10.1016/j.cub.2015.04.021 PMC5040500

[33] Bowman AR, Bruce V, Colbourn CJ, Collerton D. Compensatory shifts in visual perception are associated with hallucinations in Lewy body disorders. Cogn Res Princ Implic 2017;2:26. 28603772 10.1186/s41235-017-0063-6 PMC5442189

[34] De Deurwaerdère P, Di Giovanni G. Serotonergic modulation of the activity of mesencephalic dopaminergic systems: therapeutic implications. Prog Neurobiol 2017;151:175–236. 27013075 10.1016/j.pneurobio.2016.03.004

[35] Ciranna L. Serotonin as a modulator of glutamate- and GABA-mediated neurotransmission: implications in physiological functions and in pathology. Curr Neuropharmacol 2006;4:101–114. 18615128 10.2174/157015906776359540 PMC2430669

[36] Collerton D, Taylor JP, Tsuda I, et al. How can we see things that are not there? Current insights into complex visual hallucinations. J Conscious Stud 2016;23:195–227.

[37] Dey M, Erskine D, Singh P, et al. Does abnormal ventral visual stream function underlie recurrent complex visual hallucinations in dementia with Lewy bodies. Presented at the International DLB Conference; December 1–4, 2015; Fort Lauderdale.

[38] Duann JR, Jung TP, Kuo WJ, et al. Single-trial variability in event-related BOLD signals. Neuroimage 2002;15:823–835. 11906223 10.1006/nimg.2001.1049

[39] Violante IR, Ribeiro MJ, Edden RA, et al. GABA deficit in the visual cortex of patients with neurofibromatosis type 1: genotype-phenotype correlations and functional impact. Brain 2013;136:918–925. 23404336 10.1093/brain/aws368

[40] Harris AD, Puts NA, Anderson BA, et al. Multi-regional investigation of the relationship between functional MRI blood oxygenation level dependent (BOLD) activation and GABA concentration. PLoS One 2015;10:e0117531. 25699994 10.1371/journal.pone.0117531 PMC4336183

[41] Hall JM, Ehgoetz Martens KA, Walton CC, et al. Diffusion alterations associated with Parkinson's disease symptomatology: a review of the literature. Parkinsonism Relat Disord 2016;33:12–26. 27765426 10.1016/j.parkreldis.2016.09.026

[42] Lee WW, Yoon EJ, Lee JY, Park SW, Kim YK. Visual hallucination and pattern of brain degeneration in Parkinson's disease. Neurodegener Dis 2017;17:63–72. 27760431 10.1159/000448517

[43] Kravitz DJ, Saleem KS, Baker CI, Ungerleider LG, Mishkin M. The ventral visual pathway: an expanded neural framework for the processing of object quality. Trends Cogn Sci 2013;17:26–49. 23265839 10.1016/j.tics.2012.10.011 PMC3532569

[44] Goldman JG, Stebbins GT, Dinh V, et al. Visuoperceptive region atrophy independent of cognitive status in patients with Parkinson's disease with hallucinations. Brain 2014;137:849–859. 24480486 10.1093/brain/awt360 PMC3983409

[45] Lenka A, Jhunjhunwala RJ, Saini J, Pal PK. Structural and functional neuroimaging in patients with Parkinson's disease and visual hallucinations: a critical review. Parkinsonism Relat Disord 2015;21:683–691. 25920541 10.1016/j.parkreldis.2015.04.005

[46] Harding AJ, Broe GA, Halliday GM. Visual hallucinations in Lewy body disease relate to Lewy bodies in the temporal lobe. Brain 2002;125:391–403. 11844739 10.1093/brain/awf033

[47] Ferman TJ, Arvanitakis Z, Fujishiro H, et al. Pathology and temporal onset of visual hallucinations, misperceptions and family misidentification distinguishes dementia with Lewy bodies from Alzheimer's disease. Parkinsonism Relat Disord 2013;19:227–231. 23182311 10.1016/j.parkreldis.2012.10.013 PMC3570751

[48] Perry RH, Irving D, Blessed G, Fairbairn A, Perry EK. Senile dementia of Lewy body type: a clinically and neuropathologically distinct form of Lewy body dementia in the elderly. J Neurol Sci 1990;95:119–139. 2157823 10.1016/0022-510x(90)90236-g

[49] Collerton D, Perry E, McKeith I. Why people see things that are not there: a novel perception and attention deficit model for recurrent complex visual hallucinations. Behav Brain Sci 2005;28:737–757. 16372931 10.1017/S0140525X05000130

